# iTRAQ-Based Proteomics Identification of Serum Biomarkers of Two Chronic Hepatitis B Subtypes Diagnosed by Traditional Chinese Medicine

**DOI:** 10.1155/2016/3290260

**Published:** 2016-11-29

**Authors:** Jiankun Yang, Lichao Yang, Baixue Li, Weilong Zhou, Sen Zhong, Zhenhua Zhuang, Bin Yang, Maoshan Chen, Quansheng Feng

**Affiliations:** ^1^Chengdu University of Traditional Chinese Medicine, Chengdu 610075, China; ^2^Handan Chinese Medicine Hospital, Handan 056001, China; ^3^West China Hospital, Sichuan University, Chengdu 610041, China; ^4^Affiliated Hospital of Chengdu University of Traditional Chinese Medicine, Chengdu 610075, China; ^5^Chengdu Life Baseline Technology, Chengdu 610041, China; ^6^Department of Biochemistry and Genetics, La Trobe Institute for Molecular Science (LIMS), La Trobe University, Melbourne, VIC 3086, Australia

## Abstract

*Background.* Chronic infection with hepatitis B virus (HBV) is a leading cause of cirrhosis and hepatocellular carcinoma. By traditional Chinese medicine (TCM) pattern classification, damp heat stasis in the middle-jiao (DHSM) and liver Qi stagnation and spleen deficiency (LSSD) are two most common subtypes of CHB.* Results.* In this study, we employed iTRAQ proteomics technology to identify potential serum protein biomarkers in 30 LSSD-CHB and 30 DHSM-CHB patients. Of the total 842 detected proteins, 273 and 345 were differentially expressed in LSSD-CHB and DHSM-CHB patients compared to healthy controls, respectively. LSSD-CHB and DHSM-CHB shared 142 upregulated and 84 downregulated proteins, of which several proteins have been reported to be candidate biomarkers, including immunoglobulin (Ig) related proteins, complement components, apolipoproteins, heat shock proteins, insulin-like growth factor binding protein, and alpha-2-macroglobulin. In addition, we identified that proteins might be potential biomarkers to distinguish LSSD-CHB from DHSM-CHB, such as A0A0A0MS51_HUMAN (gelsolin), PON3_HUMAN, Q96K68_HUMAN, and TRPM8_HUMAN that were differentially expressed exclusively in LSSD-CHB patients and A0A087WT59_HUMAN (transthyretin), ITIH1_HUMAN, TSP1_HUMAN, CO5_HUMAN, and ALBU_HUMAN that were differentially expressed specifically in DHSM-CHB patients.* Conclusion.* This is the first time to report serum proteins in CHB subtype patients. Our findings provide potential biomarkers can be used for LSSD-CHB and DHSM-CHB.

## 1. Introduction

Chronic hepatitis B virus (CHB) infection is a leading cause of cirrhosis and hepatocellular carcinoma (HCC) and, in addition to morbidity and mortality, creates significant economic and social burdens [1, 2]. It is estimated that approximately 240 million people have CHB infection worldwide and CHB infection should be responsible for 650,000 cases of hepatocellular carcinoma [2, 3]. Due to the pathogenicity of CHB, early detection of CHB infection is the goal of treatment to diagnose and prevent the progression [4]. To this end, several hepatitis B virus (HBV) markers have been identified, including antigens (hepatitis B surface antigen, HBsAg; hepatitis Be antigen, HBeAg; hepatitis B core antigen, HBcAg), antibodies (hepatitis surface antibody, anti-HBs; hepatitis Be antibody, anti-HBe; hepatitis B core antibody, anti-HBc), and immunoglobulin (Ig) G and immunoglobulin M; however, unequivocal diagnosis requires more biomarkers [5].

By traditional Chinese medicine (TCM) pattern classification, CHB infected patients are accordingly classified into six subtypes [6]: (1) damp heat stasis in the middle-jiao (DHSM), (2) liver Qi stagnation and spleen deficiency (LSSD), (3) Yang deficiency of spleen and kidney (YDSK), (4) Yin deficiency of liver and kidney (YDLK), (5) blood stasis into collateral (BSIC), and (6) damp heat complicated with blood stasis (DHBS). Among them DHSM and LSSD are two most common CHB subtypes and have unique syndromes in clinic. For example, LSSD patients always have main syndromes, such as (Mi) flank pain and (Mii) abdominal distension and loose stools, and secondary symptoms, including (Si) depression and boredom, (Sii) body tired fatigue, and (Siii) pale tongue with teeth marks. DHSM patients have another two main syndromes, such as (M1) abdominal distension and (M2) yellow greasy moss, and three secondary syndromes, including (S1) nausea, being tired of the oil, and poor appetite, (S2) jaundice, bright color, and dark urine, and (S3) viscous stool foul smell. However, these syndromes are diagnosed by TCM doctors according to their experiences and the molecular biomarkers remain unclear.

Proteomics is a powerful technology recently developed to enhance our study on the diagnosis, treatment, and prevention of human diseases [7]. Among the proteomics technologies iTRAQ (isobaric Tags for Relative and Absolute Quantitation) has become popular for protein identification and quantification due to its sensitivity, accuracy, and high throughput [8]. It has been used to identify biomarker proteins for different stages of hepatitis B related diseases in patients and cellular models [9–12]. Several serum proteins have been reported to be potential biomarkers for CHB, such as actin [13], apolipoproteins A-I and A-IV [14], complement component [15], immunoglobulin related proteins [15, 16], haptoglobins *β* and *α*2 chain [14], and transferrin [17].

In this study, we employed iTRAQ combined with LC-ESI-MS/MS analyses to investigate protein biomarkers in the serum samples of two CHB subtype patients (LSSD and DHSM). Compared to healthy controls we found a number of proteins differentially expressed in both LSSD and DHSM CHB subtypes, such as actin, apolipoprotein, complement component, and immunoglobulin related proteins. In addition, we identified some proteins differentially expressed exclusively in one of LSSD and DHSM groups, such as gelsolin (GSN), likely SNC73 protein, and transient receptor potential cation channel subfamily M member 8 (TRPM8) that were found with different expression in LSSD-CHB patients only and transthyretin (TTR), tubulin, and keratin types I and II that were differentially expressed in DHSM-CHB patients only. Our findings not only validate previously reported CHB protein biomarkers but also report for the first time protein biomarkers for LSSD and DHSM CHB subtypes. The output of this study gives a valuable resource for future HBV associated studies and provides new insights of traditional Chinese medicine on molecular level.

## 2. Materials and Methods

### 2.1. Ethics Statement

This study was conducted in compliance with the Declaration of Helsinki, the ethics approval was granted by the research medical ethics committee of Chengdu University of Traditional Chinese Medicine, and signed informed consent was obtained from all participants.

### 2.2. Patients and Serum Collection

A total of 104 CHB patients were enrolled from West China Hospital, Sichuan University, and filtered with strict clinical evaluation described below. For iTRAQ proteomics analysis, we obtained blood samples from 30 LSSD-CHB patients, 30 DHSM-CHB patients, and 20 healthy controls (HCTL). For western blot analysis, 9 LSSD-CHB patients, 9 DHSM-CHB patients, and 6 HCTL participants were enrolled. Serum was collected from blood sample (4 mL) following the manufacture's protocol. Briefly, blood sample was incubated at room temperature for 2 h in vacutainer blood handling tube (Becton Dickinson, New Jersey, USA) and centrifuged for 10 min at 3,000 rpm and 4°C. Serum sample, which is the result supernatant, was transferred into a clean polypropylene tube and stored at −80°C.

### 2.3. Clinical Evaluation

The viral markers HBsAg, HBeAg, anti-HBs, anti-HBc, and anti-HBe were determined routinely in serum samples using standard procedures (AxSYM®; Abbott Laboratories, Rungis, France), as well as other molecular diagnostic markers like ALT (alanine transaminase), AST (aspartate aminotransferase), STB (serum total bilirubin), CB (conjugated bilirubin), UCB (unconjugated bilirubin), and HBV-DNA. Participants, who have hepatitis B history or HBsAg positive history for more than six months, were diagnosed as chronic HBV infection if they were positive to HBsAg and/or HBV-DNA. We used both western and Chinese medicine criteria to divide CHB patients into two groups. First, participants were satisfied with the following requirements: (1) serum HBsAg positive for over 6 months; (2) HBV-DNA positive; (3) continuous or repeated elevated serum ALT in last 12 months; (4) being 18~60 years old; (5) no planed move during the test. Then, LSSD-CHB and DHSM-CHB patients were diagnosed using the clinical symptoms mentioned before. CHB patients were diagnosed as LSSD-CHB when they met the criteria: (1) Mi and Mii; (2) Mi, Sii, and Siii; (3) Mii and Si. DHSM-CHB patients were diagnosed as follows: (1) M1 and M2; (2) M1, S1, and S2; (3) M2 and two of the secondary symptoms. We also filtered the patients when they satisfied one of the following criteria: (1) being associated with other types of hepatitis viruses or human immunodeficiency virus (HIV); (2) cirrhosis, malignancy; (3) being diagnosed with fulminant hepatitis (including acute, subacute, and chronic severe hepatitis); (4) being associated with drug or toxic liver, autoimmune hepatitis, and genetic-metabolic liver disease; (5) heart, lung, kidney, endocrine, blood, and other serious diseases; (6) pregnant women and lactating women; (7) mental disorders, in line with Chinese Classification of Mental Disorders Diagnosis (CCMD-3) standard; (8) other individuals not suitable for the cohort study.

### 2.4. Protein Preparation

Serum sample (200 *μ*L) from each patient was processed to reduce the complexity by using ProteoMiner™ Kits (Bio-Rad Laboratories, Hercules, CA, USA). Then, the sample was eluted using Lysis buffer at pH 8.5 (2M Thiourea, 7M Urea, 4% CHAPS, and 40 mM Tris-HCl), reduced using 10 mM DTT at 56°C for 1 h, and alkylated using 55 mM IAM in darkness for 1 h. After being precipitated within chilled acetone (4 × volume) at −20°C overnight, the protein sample was centrifuged at 30,000 ×g for 15 min at 4°C; the pellet was next dissolved in 500 *μ*L of 0.5 M triethylammonium bicarbonate (Applied Biosystems, Milan, Italy) and sonicated at 200 W in ice for 15 min. Finally, the samples were centrifuged again at 30,000 ×g for 15 min at 4°C, and the supernatant was quantified using Bradford Protein Assay Kit (CWBIO, Beijing, China) and stored at −80°C for subsequent analysis.

### 2.5. iTRAQ Sample Labelling, SCX Fractionation, and LC-ESI-MS/MS Analysis

Proteins isolated from 10 individuals in the same group were pooled for iTRAQ labelling. Pooled protein samples (100 *μ*g) were digested using Trypsin Gold (Promega, Madison, WI, USA) at 37°C for 16 h (protein: trypsin = 30 : 1). Digested peptides were dried by vacuum centrifugation, reconstituted in 0.5 M triethylammonium bicarbonate (Applied Biosystems, Milan, Italy), and processed 8-plex iTRAQ (Applied Biosystems) labelling following the protocols. Samples were labelled with the iTRAQ tags as follows: LSSD-CHB (113, 115, and 117), DHSM-CHB (114, 116, and 118), and HCTL (119 and 121). After being incubated at room temperature for 2 h, all the peptide mixtures were then pooled and dried by vacuum centrifugation. Strong cation exchange (SCX) chromatography was performed using the LC-20AB HPLC Pump system (Shimadzu, Kyoto, Japan), as previously described [18].

SFX fractions were resuspended in buffer A (2% ACN, 0.1% FA), followed by a centrifugation at 20,000 g for 10 min. Then, 10 *μ*L of the supernatant was loaded onto a 2 cm C18 trap column on a LC-20AD nanoHPLC (Shimadzu, Kyoto, Japan) by the autosampler and eluted onto a 10 cm analytical C18 column (inner diameter 75 *μ*m) packed in-house. At 300 nL/min the samples were loaded with buffer B (98% ACN, 0.1% FA) as the following procedural: 5% B for 1 min, a 44 min gradient from 2 to 35% B, a 2 min linear gradient to 80%, and 80% B for 4 min. Finally, the chromatographic conditions were restored in 1 min. Data acquisition was performed with an Q EXACTIVE (Thermo Fisher Scientific, San Jose, CA) coupled online to the HPLC, as described [19, 20].

### 2.6. Database Search and Protein Quantification

Database search and protein quantification were performed using Mascot (Matrix Science, London, UK; version 2.4.0). Briefly, raw data files acquired from the Orbitrap were converted into mascot generic format (MGF) files using msconvert tool of ProteoWizard (http://proteowizard.sourceforge.net/) [21]. To identify expressed proteins and quantify them, all 20 MGF files were merged and searched against UniProtHUMAN (2016_04, 152,544 sequences) database using Mascot with parameters: quantification: iTRAQ 8plex (Applied Biosystems iTRQA™ 8-plex); enzyme: trypsin; fixed modification: carboxymethyl (C), iTRAQ8plex (N-term) and iTRAQ8plex (K); variable modifications: dioxidation (M), oxidation (M), and iTRAQ8plex (Y), mass values: monoisotopic; peptide mass tolerance: ±15 ppm; fragment mass tolerance: ±20 mmu; max missed cleavages: (1) The charge states of peptides were set to +2 and +3. Specifically, an automatic decoy database search was performed in Mascot by choosing the decoy checkbox in which a random sequence of database is generated and tested for raw spectra as well as the real database. To reduce the probability of false peptide identification, only peptides at the 95% confidence interval by a Mascot probability analysis greater than “identity” were counted as identified. And each confident protein identification involves at least one unique peptide.

### 2.7. Protein Different Expression and Functional Analysis

To identify differentially expressed proteins in LSSD-CHB and DHSM-CHB compared to HCTL, we set a cut-off for fold change (>1.2) of protein abundance provided by Mascot and *p* value (<0.05) calculated by edgeR [22]. Venn diagram of up- and downregulated proteins was analyzed by InteractiVenn (http://www.interactivenn.net/) [23]. To annotate potential functions of proteins, UniProt IDs of candidate proteins were submitted to DAVID Bioinformatics Resources 6.7 (https://david.ncifcrf.gov/home.jsp) [24] and STRING v10 (http://string-db.org/) [25], Gene Ontology (GO), and KEGG pathway were selected, and we used false discovery rate (FDR) to control the results. Protein-protein interaction networks were analyzed by STRING.

### 2.8. Western Blot Analysis

Protein samples obtained from serum of 9 LSSD-CHB patients, 9 DHSM-CHB patients, and 6 healthy individuals were resolved by 12% SDS-PAGE using Miniprotean II electrophoresis unit (Bio-Rad) run at constant 120 V for 1 h and transferred to a PVDF membrane (Amersham Biosciences) under a constant voltage of 15 V for 20 min. The membranes were blocked with 5% skim milk powder in Tris-buffered saline with 0.05% Tween-20 (TTBS) for 1 h and probed in TTBS with primary antibodies (1 : 500, Santa Cruz Biotechnology, CA, USA), anti-PSMA7 (sc-166761), anti-PF4V (sc-367359), anti-PSMA6 (sc-271187), anti-SERPING1 (sc-377062), anti-ACTB (sc-8432), anti-AHSG (sc-137102), anti-CTSC (sc-74590), anti-PLTP (sc-271596), and anti-ALB (sc-46293), followed by incubation with secondary antibody (1 : 1000) for 1 h in darkness. All antibody incubations were carried out using gentle orbital shaking at room temperature. Western blots were washed five times in TTBS (5 min × 2 and 10 min × 3) after each incubation step and visualized with enhanced chemiluminescence (ECL, GE Healthcare) following the manufacturers' instructions. Band intensities on the Western blots were quantified using ImageJ (Wayne Rasband, National Institutes of Health). Albumin was used as reference to calculate the relative intensity of each protein. Then, mean ± SD values of each protein in HCTL and patients were calculated and compared using GraphPad Prism (http://www.graphpad.com/).

### 2.9. Statistical Analysis

Statistical analysis including the calculation of mean value, standard deviation (SD), and students' *t*-test was performed by using GraphPad Prism (v 6.02). The Holm-Sidak method was used to calculate the statistical significance of multiple clinical diagnostic values.

## 3. Results

### 3.1. Diagnosis of the Patients

To study serum protein biomarkers in LSSD and DHSM CHB patients, we obtained a total of 80 participants, including 30 LSSD-CHB, 30 DHSM-CHB patients, and 20 healthy volunteers. As shown in Table 1 and Table S1 in Supplementary Material available online at http://dx.doi.org/10.1155/2016/3290260, mean ages of LSSD-CHB, DHSM-CHB, and HCTL were 30, 36.8, and 35.5 years, respectively. Except missing information of three, all patients were positive to HBsAg and anti-HBc. There were two LSSD-CHB patients positive to anti-HBs, and 9 LSSD-CHB and 12 DHSM-CHB patients positive to anti-HBe. HBV-DNA levels in the blood samples of LSSD-CHB and DHSM-CHB patients were ranged from 5.12*E* + 03~1.12*E* + 08 IU/mL and 6.34*E* + 04~9.40*E* + 08 IU/mL, respectively. It is interesting that hepatitis B viral load (HBV-DNA copies) was significantly different (*p* = 0.0096) in LSSD-CHB and DHSM-CHB patients. Next, we examined ALT, AST, STB, CB, and UCB levels in the blood samples of CHB patients. Mean values of these diagnosis biomarkers in the blood samples of LSSD-CHB and DHSM-CHB patients were similar. In addition, the levels of ALT and AST remained at a high level, compared to healthy individuals [26, 27], which confirmed their CHB infection.

### 3.2. Protein Identification and Quantification by iTRAQ

**Figure 1:**
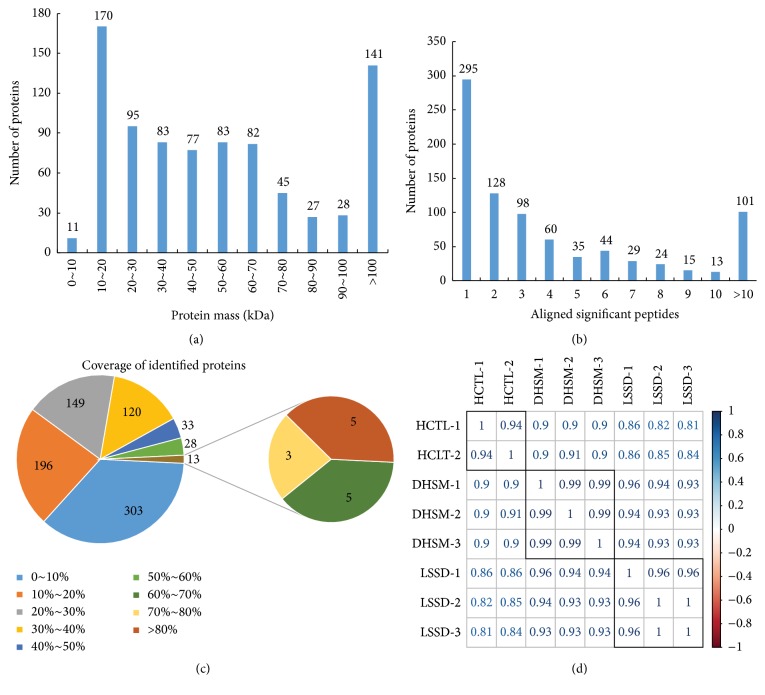
Identification and analysis of serum proteome of CHB infected patients. (a) Distribution of protein mass of identified proteins. (b) Number of peptides that match to proteins as indicated by MASCOT 2.4.0. (c) Coverage of identified proteins in CHB patient serum. (d) Correlation between samples calculated by corrplot.

Next, we quantified the serum proteins in these LSSD-CHB and DHSM-CHB patients using iTRAQ. Initially, a total of 371,034 spectra were generated by liquid chromatography coupled to mass spectrometry (LC-MS/MS) analysis. Of them, 98,243 spectra (5,591 unique peptides) were aligned to 842 proteins from 666 families. The mass distribution of identified proteins (Figure 1(a)) suggested by Mascot revealed 170 (98.69%) were above 10 kDa, of which 170 (20.19%) and 141 (16.75%) were 10 to 20 kDa and above 100 kDa, respectively. We also counted the proteins aligned with significant peptides, shown in Figure 1(b), and 547 (64.96%) proteins were aligned by two and more peptides. In addition, the distribution of protein sequence coverage is shown in Figure 1(c). Protein sequence coverage with 40~100%, 30~40%, 20~30%, 10~20%, and under 10% variation accounted for 8.79%, 14.25%, 17.70%, 23.28%, and 35.99%, respectively. In Figure 1(d), we showed correlation between two samples and found LSSD-CHB samples were closer to DHSM-CHB samples than HCTL.

### 3.3. Identification of Differentially Expressed Proteins

**Figure 2:**
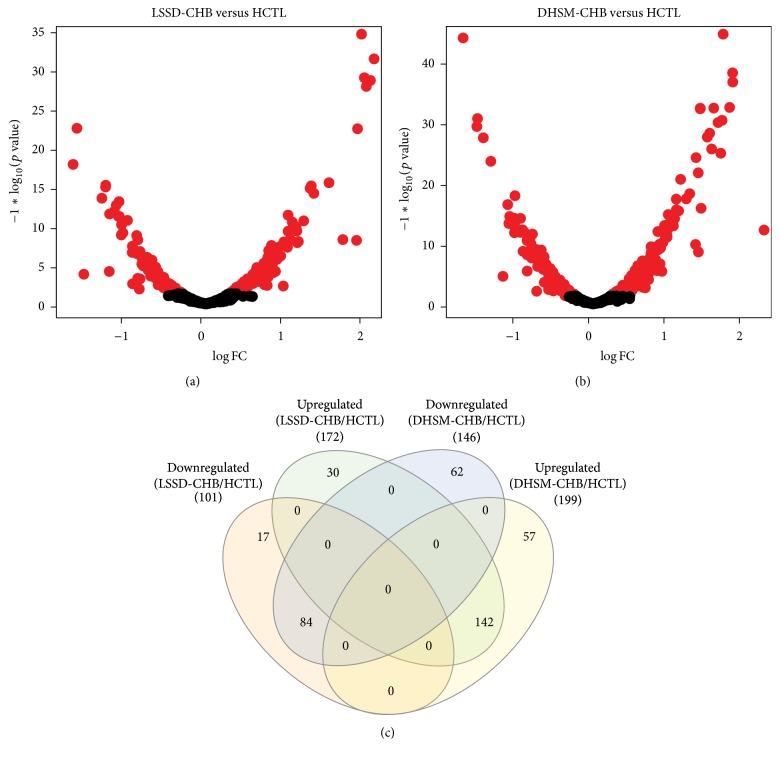
Identification of differentially expressed proteins in LSSD-CHB and DHSM-CHB patients compared with HCTL. (a) Volcano plot of differentially expressed proteins between LSSD-CHB and HCTL groups. (b) Volcano plot of differentially expressed proteins between DHSM-CHB and HCTL groups. (c) Venn diagram of up- and downregulated proteins in LSSD-CHB and DHSM-CHB groups.

Differentially expressed proteins were defined as those showed greater than 1.2-fold change in relative abundance and a *p* value < 0.05. Compared to HCTL we identified a total of 392 proteins differentially expressed (Table S2), of which 273 were identified in LSSD-CHB group and 345 in DHSM-CHB group. As shown in the volcano plots, we identified 172 upregulated and 101 downregulated proteins in LSSD-CHB group (Figure 2(a)) and 199 upregulated and 146 downregulated proteins in DHSM-CHB group (Figure 2(b)), compared to HCTL group. Venn diagram (Figure 2(c)) revealed LSSD-CHB and DHSM-CHB shared 142 upregulated and 84 downregulated proteins; 30 and 57 proteins were exclusively upregulated in LSSD-CHB and DHSM-CHB, respectively; 17 and 62 proteins were exclusively downregulated in LSSD-CHB and DHSM-CHB, respectively; and no protein was identified with upregulation in one CHB subtype but with downregulation in another.

### 3.4. Potential Biomarkers for CHB

The identification of proteins differentially expressed in LSSD-CHB and DHSM-CHB groups relative to the HCTL group was of interest as these could provide leads for potentially useful diagnostic and prognostic biomarkers. First, we examined those 142 commonly upregulated and 84 commonly downregulated proteins. As shown in Table 2, the largest upregulated protein family was immunoglobulin related protein, showing 20 upregulated and 3 downregulated proteins identified. In clinical immunology, levels of immunoglobulins especially IgG can be used to characterize viral hepatitis in patients [28, 29]. Four IgG subclasses (IgG1 to IgG4) differ in their heavy chain constant regions and have different effects on virus-cell fusion inhibition, virus neutralization, and overall course of infection, as have been reported for various viruses including HIV [30] and HBV [31]. Highly expressed proteins encoding heavy chains for immunoglobulins including IGHG1, IGHG3, IGHG4, and IGH@ have been reported with upregulation in HBV [32, 33] and HCC patients [34]. Other upregulated protein families such as heat shock protein, histone, ras-related protein, and von Willebrand factor identified in current study have also been reported in patients infected by HBV or hepatitis C virus (HCV) [35–38]. The largest downregulated protein family was complement, 15 complement proteins downregulated in LSSD-CHB with 0.82- to 0.37-fold change and in DHSM-CHB with 0.77- to 0.31-fold change. Other protein families like insulin-like growth factor binding protein and serum amyloid protein were also decreased in CHB patients in comparison to HCTL group. In addition, several known upregulated proteins from other families in patients infected by HBV or HCV (Table 2), such as apolipoproteins (APOA2, APOB, and APOB-variant) [39, 40], A2M (alpha-2-macroglobulin) [41], alpha-actinin-3 (ACTN3) [42, 43], vimentin (VIM) [38], and putative uncharacterized proteins (DKFZp686N02209 and DKFZp686I04196) [34, 44, 45], were identified in LSSD-CHB and DHSM-CHB groups. The different expression of proteins in the serum of CHB patients indicates they may have functions in response of HBV and CHB processing and can be used as biomarkers in clinical diagnosis.

**Figure 3:**
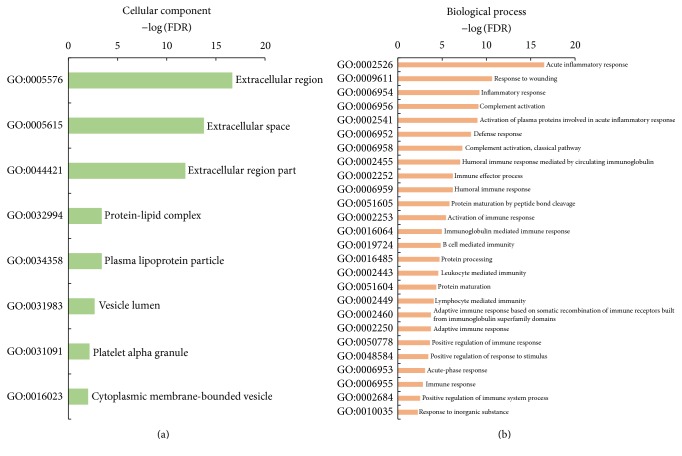
Gene Ontology annotation for shared differentially expressed proteins in LSSD- and DHSM-CHB groups: (a) cellular component, (b) biological process.

We next analyzed the potential functions of commonly differentially expressed serum proteins in LSSD-CHB and DHSM-CHB groups using DAVID Bioinformatics Resources 6.7 [24] and STRING v10 [25]. Cellular component annotation (Figure 3(a)) showed 63 and 7 proteins were “extracellular region” (GO: 0005576, GO: 0005615, and GO: 0044421) and “lipids” (GO: 0032994 and GO: 0034358), respectively. However biological process annotation (Figure 3(b)) showed most of the differentially expressed proteins associated with immune response, including “acute inflammatory response” (GO: 0002526), “response to wounding” (GO: 0009611), “inflammatory response” (GO: 0006954), “complement activation” (GO: 0006956), “defense response” (GO: 0006952), “humoral immune response mediated by circulating immunoglobulin” (GO: 0002455), “immune effector process” (GO: 0002252), “B cell mediated immunity” (GO: 0019724), and “activation of immune response” (GO: 0002253). It has been well studied that immunological events are necessary to control hepatitis B virus (HBV) infection [46, 47]. In addition, KEGG pathway analysis also showed differentially expression proteins function mainly in the pathways of “complement and coagulation cascades” (hsa04610), “systemic lupus erythematosus” (hsa05322), “focal adhesion” (hsa04510), and “viral carcinogenesis” (hsa05203). Overall, differentially expressed proteins in both LSSD-CHB and DHSM-CHB groups have potential ability to be used as biomarkers.

### 3.5. Dysregulated Proteins Detected Exclusively in LSSD-CHB and DHSM-CHB

Next, we examined differentially expressed proteins exclusively in LSSD-CHB and DHSM-CHB groups. A total of 30 upregulated and 17 downregulated proteins were specifically identified in LSSD-CHB patient serum samples (Table 3). Among them 11 upregulated immunoglobulin related proteins, gelsolin (GSN), serum paraoxonase/lactonase 3 (PON3), likely SNC73 protein, transient receptor potential cation channel subfamily M member 8 (TRPM8), and several uncharacterized proteins (DKFZp686M08189, DKFZp686C02220, and DKFZp686K04218) attracted our attention due to their high abundance. Serum PON3 concentrations have been reported to increase in patients with CHB or cirrhosis and showed significant direct correlations with the degree of periportal abnormalities including fibrosis and with serum FAS (a marker of antiapoptosis) concentrations [48]; however, serum gelsolin level has been reported to reduce significantly in patients with acute liver failure (47%), myocardial infarction (69%), sepsis (51%), and myonecrosis (66%) [49]. Among the specifically downregulated serum proteins in LSSD-CHB patients fibulin-1 (FBLN1) is a tumor suppressor in hepatocellular carcinoma [50]. Proteins specifically differentially expressed in LSSD-CHB patients were predicted to function mainly in biological processes of “protein activation cascade” (GO: 0072376), “regulation of response to wounding” (GO: 1903034), “blood coagulation, fibrin clot formation” (GO: 0072378), “negative regulation of response to stimulus” (GO: 0048585), and “acute-phase response” (GO: 0006953).

We also identified 57 upregulated and 62 downregulated proteins exclusively in DHSM-CHB patients (Table 4). Two IGL@ proteins (Q6GMX4_HUMAN and Q6PIQ7_HUMAN) were specifically upregulated in DHSM-CHB patients with 1.27-fold change. Transthyretin (TTR), upregulated 1.34-fold in DHSM-CHB, can be induced by hepatitis C virus and activate TGF-*β* signaling pathway with furin [51]. Interestingly, we found three members of tubulin (TUBA4A, TUBB1, and TUBB8) were upregulated only in DHSM-CHB patients compared with HCTL. Although there are few reports about tubulin and HBV, it is well known that 42 kDa tubulin alpha-6 chain fragment in well-differentiated hepatocellular carcinoma tissues is from patients infected with HCV [52]. In addition, we found actinin, alpha 1 (ACTN1), which can directly interact with HCV [53], GAPDH, which can bind to the HBV posttranscriptional regulatory element [54], and polymeric immunoglobulin receptor (PIGR), the main transporter of IgA [55], were upregulated in DHSM-CHB but not in LSSD-CHB. Among DHSM-CHB specifically downregulated proteins we identified three members of keratin type I (KRT9, KRT10, and KRT14) and another three members of keratin type II (KRT1, KRT2, and KRT6B). Although there is no evidence showing relation between these six keratin proteins with CHB or other liver diseases, variant keratins are associated with progression of fibrosis during chronic hepatitis C infection [56]. Differentially expressed proteins exclusively detected in DHSM-CHB patients were predicted to be involved in the biological processes of “immune system process” (GO: 0002376), “response to stress” (GO: 0006950), “defense response” (GO: 0006952), “immune response” (GO: 0006955), and “single-organism metabolic process” (GO: 0044710).

Compared to HCTL group up- and downregulated proteins exclusively in LSSD-CHB and DHSM-CHB patients showed their potential ability of being biomarkers for these two subtypes of HBV induced CHB. Some of them have been reported in other studies; however, more experiments need to be performed to investigate their functions and validate their specificity and accuracy in clinical trials.

### 3.6. Validation of the Quantitative Proteomic Analysis

**Figure 4:**
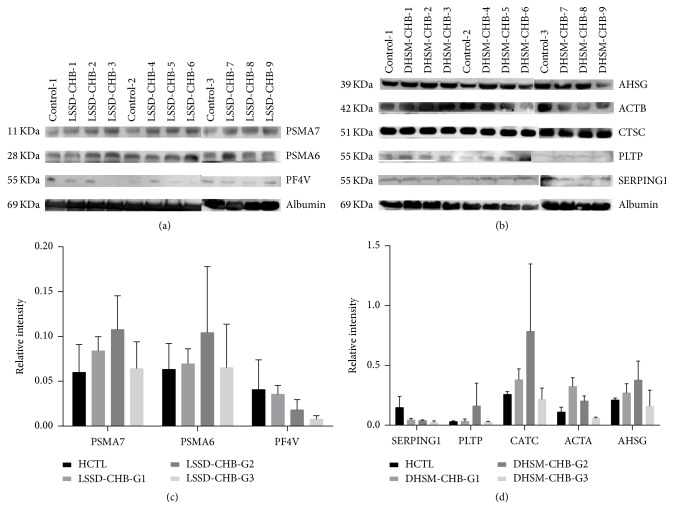
Western blot analysis. (a) Western blot analysis for PSMA7, PSMA6, and PF4 V in LSSD-CHB patients. (b) Western blot analysis for AHSG, ACTB, CTSC, PLTP, and SERPING1 in DHSM-CHB patients. Box plots of the relative intensity of candidate proteins in LSSD-CHB (c) and DHSM-CHB (d) groups.

To validate the results obtained by proteomics analysis, eight randomly selected proteins and internal control albumin with altered expression profile were monitored by western blotting in an independent group of samples. Figures 4(a) and 4(b) showed the western blots for eight proteins and internal control albumin. PSMA6 (20S proteasome alpha6), PSMA7 (20S proteasome alpha7/alpha8) were upregulated and PF4 V (platelet factor 4 variant) was downregulated in LSSD-CHB group compared to HCTL (Figure 4(c)). Except SERPING1 (plasma protease C1 inhibitor), AHSG (fetuin-A), ACTB (actin), CTSC (cathepsin C), and PLTP (phospholipid transfer protein) were upregulated in the serum of DHSM-CHB patients (Figure 4(d)). Although the difference between patients and healthy participants was not significant by western blotting analysis, their regulations in patients and healthy group were consistent with iTRAQ. The original images of western blots (see Figure S1) might contain some differences due to brightness and contrast settings.

## 4. Discussion

Quantitation of serum or plasma proteins using comparative proteomics has recently been suggested as a suitable approach for the detection of liver disease biomarkers [17, 57–59]. The iTRAQ technology has been proposed as a powerful alternative to common tools (e.g., ELISA) and a flurry of applications emerged in the literature.

**Figure 5:**
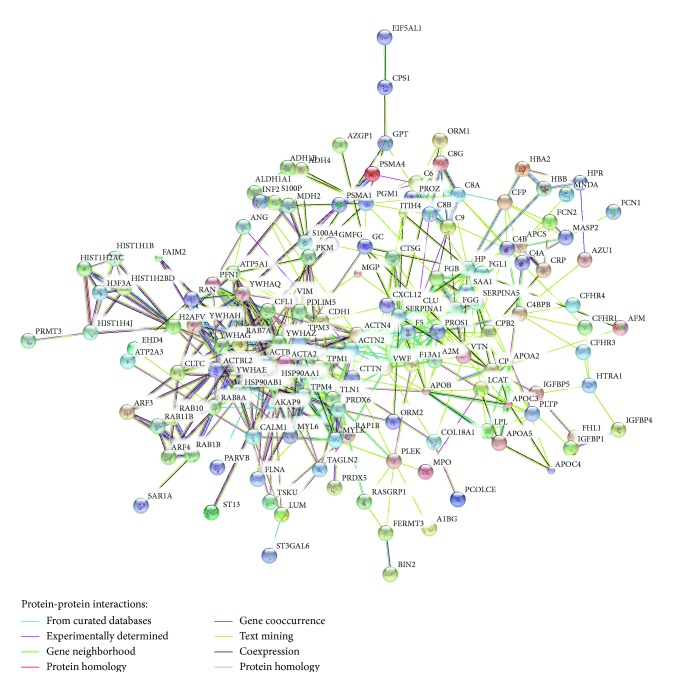
Protein-protein interaction network of commonly differentially expressed proteins in LSSD-CHB and DHSM-CHB patients compared with HCTL group.

In this study, iTRAQ LC–MS/MS proteomics was used to detect serum protein as biomarkers of LSSD-CHB and DHSM-CHB patients. We compared the proteomics profile of LSSD-CHB and DHSM-CHB patients with healthy individuals and indicated 142 upregulated and 84 downregulated proteins shared by these two CHB subtype diseases. Protein-protein interaction network (Figure 5) showed several significant proteins might function in response to HBV, such as actins (ACTA2, ACTB, ACTBL2, ACTN3, and ACTN4), apolipoproteins (APOA2, APOA5, APOB, APOC3, and APOC4), heat shock proteins (HSP90AA1 and HSP90AB1), and proteasome subunit proteins (PSMA1 and PSMA4). It has been reported that HBV core proteins can interact with the C-terminal region of actin-binding protein [60] and HBV X protein (HBx) can block filamentous actin bundles by interaction with eEF1A1 (eukaryotic translation elongation factor 1 alpha 1) [61]. In addition, ACTA2 is a marker of hepatitis stellate cells and correlated significantly with necroinflammatory grades and fibrotic stages in CHB or CHC [13]. Apolipoproteins are supposed to enhance the infectivity of hepatitis virus during the infection [39, 62] and are identified to interact with HBx as well [63]. Among the apolipoproteins APOA2 is a considerable biomarker because its expression is increased on both mRNA and protein levels in CHB patients [14, 64]. HBx protein also interacts with heat shock proteins and enhances HBx-mediated apoptosis [65]. A HBV-specific peptide (TVATAMG) is associated with heat shock protein and has potential for engineering tumor vaccines against hepatocellular carcinoma and chronic HBV infection [66]. Heat shock proteins like HSP27, HSP90, and GRP78 are upregulated in HBV related hepatocellular carcinoma, associated with vascular invasion and intrahepatic metastasis and have potential to be prognosis markers [67, 68]. Commonly downregulated complement proteins are important mediators of inflammation and contribute to the regulation of the immune response. C4, a predisposing factor to autoimmune chronic active hepatitis [69], is expressed lowly in chronic hepatitis C patient compared to that in controls [70]. Low serum levels of complement in viral hepatitis are associated with high titers of hepatitis-associated antigen [71]. It is said that complement proteins are related to hepatitis B vaccine and C4AQ0 (mutant C4) probably contribute to inefficient complement activation and failure of B cells to secret anti-HBs [72]. Our results confirmed the potential of previously reported proteins in diagnosis of patients infected by HBV.

**Figure 6:**
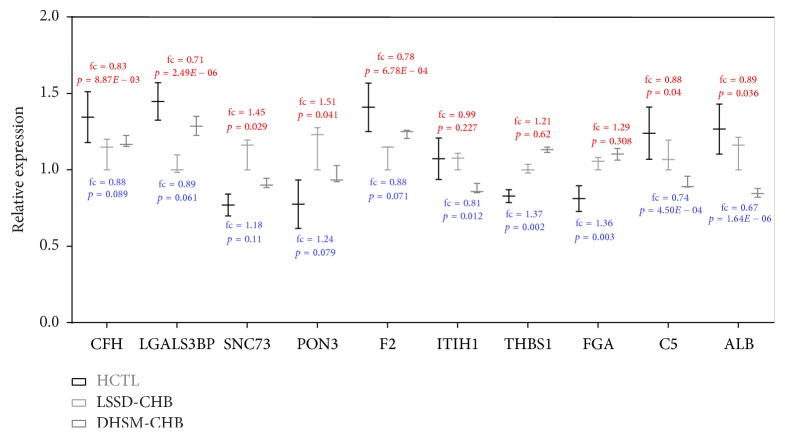
Top 5 highly expressed proteins differentially expressed in LSSD-CHB and DHSM-CHB compared to HCTL. Fold changes (fc) and *p* values (*p*) of them were shown in red and blue for LSSD-CHB and DHSM-CHB, respectively.

LSSD-CHB and DHSM-CHB are two subtypes of CHB according to traditional Chinese medicine pattern classification. In this study we identified 47 and 119 differentially expressed proteins exclusively in LSSD-CHB and DHSM-CHB, respectively, which could be used as biomarkers for LSSD-CHB and DHSM-CHB patients. We showed top 5 highly expressed proteins with different expression in LSSD-CHB and DHSM-CHB patients compared to HCTL group in Figure 6. Using relative expression ratio calculated by MASCOT we found mean expression levels of some proteins were close in LSSD-CHB and DHSM-CHB but with different *p* values, such as CFH (complement factor H), F2 (prothrombin), and FGA (fibrinogen alpha chain). As we know, prothrombin time is one of the markers of liver test; it is usually lower in HBV infected patients than in healthy people and a good marker for liver fibrosis [73, 74]. CFH functions as a cofactor in the inactivation of C3b by factor I [75], which can interact with IgG and is moderately depressed in the serum of patients with viral hepatitis [71]. FGA has a major function in hemostasis as one of the primary components of blood clots [76]. Fibrinogen-like protein 2 (FGL2) has been identified as a potential biomarker for severity of CHC infection [77]. Other proteins also have been reported to be associated with HBV infection. LGALS3BP (lectin galactoside-binding soluble 3 binding protein isoform 1) were downregulated in LSSD-CHB patients (fc = 0.71, *p* value = 2.49*E* − 06) and DHSM-CHB patients (fc = 0.89, *p* value = 0.061). Previous studies about LGALS3BP in CHB and HCC found its different expression on transcriptional level [78], while in current study we identified its protein was differentially expressed in CHB patients and had the potential to be a good marker for LSSD-CHB subtype. PON3 (serum paraoxonase/lactonase 3), which was upregulated exclusively in LSSD-CHB, might play a hepatoprotective role against histological alterations and hepatic cell apoptosis leading to liver disease [48].

DHSM-CHB specifically differentially expressed proteins like ITIH1 (inter-alpha-trypsin inhibitor heavy chain H1), THBS1 (thrombospondin-1), C5 (Complement C5), and ALB (albumin) have been also reported in hepatitis viral related diseases. The expression level of ITIH1 in HCTL group was similar to that in LSSD-CHB patients (fc = 0.99, *p* value = 0.227) but was downregulated significantly in DHSM-CHB patients (fc = 0.81, *p* value = 0.012). The low expression of ITIH1 indicated it can be used to differ DHSM-CHB from LSSD-CHB. In addition, it has been experimented to be downregulated in HCV infected patients [79] and hepatitis C associated hepatocellular carcinoma patients [80]. It is reported that HCV viral proteins act directly or indirectly on THBS1 in TGF-*β* pathway [81]. By noninvasive imaging the gene expression of THBS1 was upregulated in liver cancer [82]. Interestingly, ALB has been reported as an important factor to score the risk of HCC in CHB patients [83]. In our study, ALB was downregulated in both CHB subtypes but significantly exclusively in DHSM-CHB. Our results confirmed its different expression in CHB patients and revealed that the criteria of ALB expression in CHB patients required more patients and experiments.

Due to the fact that hepatitis B viral load in DHSM-CHB patients was significantly higher than that in LSSD-CHB patients, we assume HBV-DNA might be related to CHB patients with different syndromes and it requires further experiments. To our knowledge, this study appears to be the first iTRAQ based approach aimed at identifying leads for potential useful biomarkers of patients of CHB subtypes. The candidates identified in this study await rigorous clinical validation using large cohorts of patient samples and more experimental function analysis.

## Supplementary Material

Liver function test and hepatitis B test were performed for all the volunteers in this study and the results can be seen in Table S1. Using iTRAQ proteomics technology, we identified a total of 842 proteins in LSSD-CHB, DHSM-CHB patients and healthy individuals. Compared to healthy individuals, we identified a total of 392 proteins differentially expressed in these two CHB subtypes, of which they shared 142 up-regulated and 84 down-regulated proteins. The full list of differentially expressed proteins and the statistical values can be seen in Table S2. Further, we used western blotting analysis to validate the expression of differentially expressed proteins and the original images of western can be seen in Figure S1.

## Figures and Tables

**Table 1:** Clinical diagnosis of patients who participated in this study.

Diagnosis	Unit	LSSD-CHB (*n* = 30)	DHSM-CHB (*n* = 30)	HCTL (*n* = 20)	*p* value
*Sex*					
Male		24	15	10	
Female		6	15	10	

*Age*	Years	17~56	18~60	24~56	
Mean age		30.8	36.83	36.15	
Standard deviation (SD)		10.526	11.885	11.554	

*Hepatitis B surface antigen (HBsAg)*					
Positive		28	29		
Negative		0	0		

*Hepatitis surface antibody (anti-HBs)*					
Positive		2	0		
Negative		25	29		

*Hepatitis Be antigen (HBeAg)*					
Positive		21	19		
Negative		7	9		

*Hepatitis Be antibody (anti-HBe)*					
Positive		9	12		
Negative		19	16		

*Hepatitis B core antibody (anti-HBc)*					
Positive		28	29		
Negative		0	0		

*Alanine transaminase (ALT)*	IU/L	13.8~627	34~673		1.0
Mean ALT level		190.153	187.393		
SD		161.231	177.618		

*Aspartate aminotransferase (AST)*	IU/L	26.5~345	28~556		0.9999
Mean AST		96.74	142.427		
SD		71.699	153.539		

*Serum total bilirubin (STB)*	umol/L	10.1~48.63	6.5~109.5		1.0
Mean STB		19.208	20.643		
SD		8.926	18.127		

*Conjugated bilirubin (CB)*	umol/L	2.4~16.4	2.3~99.2		1.0
Mean CB		6.923	9.408		
SD		3.542	17.208		

*Unconjugated bilirubin (UCB)*	umol/L	6~34.13	3.3~29.3		1.0
Mean UCB		12.284	11.182		
SD		5.951	4.921		

*HBV-DNA*	IU/mL	5.12*E* + 03~1.12*E* + 08	6.34*E* + 04~9.40*E* + 08		0.0096
Mean HBV-DNA		2.521*E* + 07	5.655*E* + 07		
SD		3.221*E* + 07	1.717*E* + 08		

**Table 2:** Commonly up- and downregulated proteins in LSSD-CHB and SSDHS-CHB groups (part).

Family	UniProt_ID	Gene_name	Description	Number of significant matches	Number of significant sequences	emPAI	LSSD-CHB versus HCTL		SSDHS-CHB versus HCTL	
							FC^a^	*p* value^b^	FC^a^	*p* value^b^
Immunoglobulin related proteins	A0A087WV47_HUMAN	IGHG1	Ig gamma-1 chain C region	1541	14	5.37	1.84	6.30*E* − 05	1.47	2.71*E* − 04
	A0A087WYC5_HUMAN	IGHG1	Ig gamma-1 chain C region	1501	14	4.82	1.87	3.51*E* − 05	1.48	1.69*E* − 04
	A0A087X1C7_HUMAN	IGHG1	Ig gamma-1 chain C region	1511	13	4.79	1.84	6.12*E* − 05	1.47	2.60*E* − 04
	A0A087WXL8_HUMAN	IGHG3	Ig gamma-3 chain C region	739	11	2.07	1.84	5.45*E* − 05	1.47	1.12*E* − 04
	A0A0G2JPD4_HUMAN	IGHG4	Ig gamma-4 chain C region (fragment)	31	6	1.56	1.69	6.47*E* − 04	1.24	4.12*E* − 02
	KV112_HUMAN		Ig kappa chain V-I region Kue	2	2	0.53	1.82	1.39*E* − 03	1.51	9.53*E* − 04
	KV311_HUMAN		Ig kappa chain V-III region IARC/BL41	2	1	0.49	1.61	5.86*E* − 03	1.28	1.64*E* − 02
	LV302_HUMAN		Ig lambda chain V-III region LOI	25	3	4.01	1.73	1.75*E* − 04	1.35	8.79*E* − 04
	LV301_HUMAN		Ig lambda chain V-III region SH	6	1	0.61	1.99	1.59*E* − 07	1.56	5.32*E* − 07
	S6B2B6_HUMAN		IgG H chain	384	3	0.6	2.05	1.06*E* − 04	1.57	9.46*E* − 04
	S6BAM6_HUMAN		IgG H chain	389	4	0.9	2.05	4.60*E* − 05	1.56	5.55*E* − 04
	S6BGE0_HUMAN		IgG H chain	394	4	1.13	2.04	4.84*E* − 05	1.56	6.39*E* − 04
	S6C4S4_HUMAN		IgG H chain	384	3	0.73	2.05	9.63*E* − 05	1.57	5.91*E* − 04
	S6C4R7_HUMAN		IgG L chain	257	6	6.68	1.53	2.61*E* − 02	1.27	4.54*E* − 02
	Q6GMX6_HUMAN	IGH@	IGH@ protein	1524	14	6.04	1.83	6.27*E* − 05	1.46	3.15*E* − 04
	A0A0F7TC28_HUMAN	IGHV4-4	IGHV4-4 protein (fragment)	3	2	1.27	1.49	9.67*E* − 03	1.24	2.27*E* − 02
	Q5FWF9_HUMAN	IGL@	IGL@ protein	143	6	6.03	1.60	1.35*E* − 02	1.32	2.83*E* − 02
	Q6PIK1_HUMAN	IGL@	IGL@ protein	197	5	4.45	1.59	1.37*E* − 02	1.29	4.62*E* − 02
	Q9UL84_HUMAN		Myosin-reactive immunoglobulin heavy chain variable region (fragment)	6	3	1.69	1.52	1.58*E* − 02	1.46	2.04*E* − 04
	Q9UL79_HUMAN		Myosin-reactive immunoglobulin light chain variable region (fragment)	2	1	0.57	0.65	6.56*E* − 04	0.50	1.93*E* − 12
	A0A0C4DH33_HUMAN	IGHV1-24	Protein IGHV1-24 (fragment)	5	2	0.84	0.82	5.43*E* − 03	0.81	1.38*E* − 02
	A0A0A0MT74_HUMAN	IGKV1-16	Protein IGKV1-16 (fragment)	5	1	0.9	1.58	3.55*E* − 03	1.26	3.22*E* − 02
	A0A075B6I9_HUMAN	IGLV7-46	Protein IGLV7-46 (fragment)	31	3	1.41	1.54	9.85*E* − 03	1.36	2.46*E* − 03

Complement	B7Z1F8_HUMAN		cDNA FLJ53025, highly similar to complement C4-B	2230	12	56.68	0.78	1.27*E* − 03	0.77	2.33*E* − 03
	B4E3S6_HUMAN		cDNA FLJ58413, highly similar to complement component C7	63	10	1.74	0.55	8.69*E* − 10	0.50	8.03*E* − 15
	A8K2T4_HUMAN		cDNA FLJ78207, highly similar to human complement protein component C7 mRNA	86	15	1.31	0.55	1.62*E* − 09	0.50	2.39*E* − 13
	B2RA39_HUMAN		cDNA, FLJ94686, highly similar to *Homo sapiens* complement factor H-related 5 (CFHL5), mRNA	35	5	0.51	0.54	7.03*E* − 12	0.57	1.44*E* − 11
	CO4A_HUMAN	C4A	Complement C4-A	5889	65	21.28	0.79	2.87*E* − 03	0.75	7.70*E* − 04
	CO4B_HUMAN	C4B	Complement C4-B	5983	66	23.03	0.79	2.95*E* − 03	0.74	5.91*E* − 04
	A0A024R035_HUMAN	C9	Complement component 9, isoform CRA_a	225	17	5.12	0.67	1.36*E* − 05	0.54	6.61*E* − 13
	CO6_HUMAN	C6	Complement component C6	92	12	0.83	0.50	3.34*E* − 12	0.48	4.06*E* − 15
	CO8A_HUMAN	C8A	Complement component C8 alpha chain	66	8	1.51	0.66	1.94*E* − 06	0.53	8.36*E* − 15
	F5GY80_HUMAN	C8B	Complement component C8 beta chain	133	16	3.28	0.65	6.59*E* − 07	0.61	1.07*E* − 09
	CO8G_HUMAN	C8G	Complement component C8 gamma chain	41	8	6.86	0.61	8.80*E* − 08	0.50	3.38*E* − 14
	B1AKG0_HUMAN	CFHR1	Complement factor H-related protein 1	216	6	4.26	0.66	1.30*E* − 06	0.75	4.80*E* − 04
	FHR3_HUMAN	CFHR3	Complement factor H-related protein 3	45	6	1.19	0.82	7.05*E* − 03	0.77	2.36*E* − 03
	FHR4_HUMAN	CFHR4	Complement factor H-related protein 4	31	3	0.41	0.37	3.90*E* − 23	0.31	1.62*E* − 44
	A0A0S2Z4I5_HUMAN	CFP	Complement factor properdin isoform 1 (fragment)	13	2	0.17	0.58	2.21*E* − 11	0.72	2.90*E* − 05

Apolipoprotein	APOA2_HUMAN	APOA2	Apolipoprotein A-II	421	2	1.84	1.48	4.64*E* − 02	1.26	3.72*E* − 02
	A0A0B4RUS7_HUMAN	APOA5	Apolipoprotein A-V, isoform CRA_a	15	7	1	0.73	2.03*E* − 05	0.62	2.95*E* − 08
	E1B4S9_HUMAN	APOB	Apolipoprotein B (fragment)	129	7	10.94	1.97	1.23*E* − 05	1.53	9.70*E* − 05
	C0JYY2_HUMAN	APOB	Apolipoprotein B (including Ag(X) antigen)	5227	215	21.89	1.84	2.08*E* − 04	1.44	1.07*E* − 03
	Q59HB3_HUMAN		Apolipoprotein B variant (fragment)	1661	69	16.33	1.85	2.79*E* − 04	1.41	1.40*E* − 03
	B0YIW2_HUMAN	APOC3	Apolipoprotein C-III	577	5	8.48	0.66	1.44*E* − 05	0.55	3.02*E* − 09
	APOC4_HUMAN	APOC4	Apolipoprotein C-IV	26	5	3.84	0.80	1.35*E* − 03	0.60	8.22*E* − 09

Histone	H15_HUMAN	HIST1H1B	Histone H1.5	4	2	0.35	2.02	8.32*E* − 06	1.72	1.02*E* − 04
	A0A024R017_HUMAN	HIST1H2AC	Histone H2A	21	4	2.27	4.67	1.78*E* − 28	3.63	4.54*E* − 33
	C9J0D1_HUMAN	H2AFV	Histone H2A	13	3	1.11	4.84	3.09*E* − 29	3.73	2.99*E* − 37
	A0A024QZZ7_HUMAN	HIST1H2BD	Histone H2B	12	4	1.15	4.58	1.39*E* − 29	3.38	6.00*E* − 31
	B2R4P9_HUMAN	H3F3A	Histone H3	9	3	0.88	4.98	5.44*E* − 32	3.72	9.13*E* − 39
	Q5TEC6_HUMAN	HIST2H3PS2	Histone H3	4	3	0.6	2.59	1.10*E* − 08	1.87	3.39*E* − 06
	B2R4R0_HUMAN	HIST1H4J	Histone H4	39	5	13.27	4.47	3.83*E* − 35	3.41	3.84*E* − 45

Heat shock protein	A0A024RD80_HUMAN	HSP90AB1	Heat shock protein 90 kDa alpha (cytosolic), class B member 1, isoform CRA_a	12	6	0.31	1.46	3.59*E* − 02	1.51	2.77*E* − 05
	HS90A_HUMAN	HSP90AA1	Heat shock protein HSP 90-alpha	28	10	0.55	1.58	5.45*E* − 03	1.65	3.81*E* − 07

Insulin-like growth factor binding protein	C1K3N3_HUMAN	IGFBP1	Insulin-like growth factor binding protein 1 (fragment)	3	2	0.22	0.61	2.62*E* − 07	0.60	5.40*E* − 09
	A0A024R1U8_HUMAN	IGFBP4	Insulin-like growth factor binding protein 4, isoform CRA_a	2	1	0.1	0.66	8.52*E* − 06	0.66	1.00*E* − 06
	A0A024R433_HUMAN	IGFBP5	Insulin-like growth factor binding protein 5, isoform CRA_a	3	2	0.18	0.83	3.37*E* − 03	0.80	4.98*E* − 02

Ras-related protein	A0A024R7I3_HUMAN	RAB8A	RAB8A, member RAS oncogene family, isoform CRA_a	11	4	1.08	1.75	1.87*E* − 03	1.73	9.62*E* − 06
	A0A024RB87_HUMAN	RAP1B	RAP1B, member of RAS oncogene family, isoform CRA_a	10	4	1.66	1.85	8.55*E* − 05	1.96	7.63*E* − 11
	RAB10_HUMAN	RAB10	Ras-related protein Rab-10	9	3	0.71	1.74	2.50*E* − 03	1.72	2.60*E* − 05
	RB11B_HUMAN	RAB11B	Ras-related protein Rab-11B	5	4	0.73	1.91	3.03*E* − 05	1.57	1.03*E* − 05
	RAB1B_HUMAN	RAB1B	Ras-related protein Rab-1B	12	6	1.5	1.73	2.07*E* − 03	1.73	7.38*E* − 06
	RB27B_HUMAN	RAB27B	Ras-related protein Rab-27B	5	4	0.7	1.70	1.33*E* − 03	1.59	2.26*E* − 05
	RAB7A_HUMAN	RAB7A	Ras-related protein Rab-7a	16	6	1.94	1.60	2.16*E* − 02	1.66	1.48*E* − 04

Serum amyloid	D3DQX7_HUMAN	SAA1	Serum amyloid A protein	59	5	9.88	0.53	2.58*E* − 13	0.40	3.36*E* − 24
	SAA1_HUMAN	SAA1	Serum amyloid A-1 protein	40	4	4.99	0.48	1.21*E* − 15	0.38	4.55*E* − 28
	SAA2_HUMAN	SAA2	Serum amyloid A-2 protein	24	4	4.99	0.48	7.27*E* − 16	0.36	3.08*E* − 31
	SAMP_HUMAN	APCS	Serum amyloid P-component	228	8	15.23	0.83	8.13*E* − 03	0.70	4.45*E* − 05

von Willebrand factor	L8E853_HUMAN	VWF	von Willebrand factor	189	38	0.98	1.68	2.69*E* − 03	1.41	9.67*E* − 04
	VWF_HUMAN	VWF	von Willebrand factor	186	38	0.93	1.67	3.05*E* − 03	1.41	7.94*E* − 04

^a^Fold change provided by MASCOT.

^b^ *p* values calculated by edgeR to show the significance of different expression.

**Table 3:** Up- and downregulated serum proteins specifically in LSSD-CHB.

UniProt_ID	Gene_name	Description	Number of significant matches	Number of significant sequences	emPAI	FC^a^	*p* value^b^
*Upregulated*							
A0A024R6I9_HUMAN	SERPINA4	Serpin peptidase inhibitor, clade A (alpha-1 antiproteinase, antitrypsin), member 4, isoform CRA_a	79	12	3.79	1.78	5.24*E* − 05
A0A075B6K3_HUMAN	IGLV2-11	Protein IGLV2-11 (fragment)	8	1	0.9	1.43	4.94*E* − 02
A0A075B6K6_HUMAN	IGLV4-3	Protein IGLV4-3	4	1	0.51	1.54	1.23*E* − 02
A0A075B6N7_HUMAN	IGHA2	Ig alpha-2 chain C region (fragment)	80	6	1.94	1.44	4.71*E* − 02
A0A0A0MS51_HUMAN	GSN	Gelsolin	146	16	2.42	1.51	2.86*E* − 02
A0A0B4J1V4_HUMAN	IGHV1-46	Protein IGHV1-46 (fragment)	2	1	0.23	1.44	4.22*E* − 02
A0N7J6_HUMAN		REV25-2 (fragment)	2	2	0.47	1.92	3.69*E* − 03
A0N8J1_HUMAN		V(k)3 sequence of NG9 gene from fetal liver DNA (fragment)	6	1	1.1	1.49	1.49*E* − 02
CPN2_HUMAN	CPN2	Carboxypeptidase N subunit 2	94	11	2.09	1.48	1.88*E* − 02
D3DNU8_HUMAN	KNG1	Kininogen 1, isoform CRA_a	91	14	3.3	1.50	1.14*E* − 02
FETUA_HUMAN	AHSG	Alpha-2-HS-glycoprotein	57	3	0.64	1.70	1.36*E* − 03
FETUB_HUMAN	FETUB	Fetuin-B	9	3	0.29	1.65	6.32*E* − 04
HV208_HUMAN		Ig heavy chain V-II region SESS	2	1	0.21	2.27	1.25*E* − 08
ITB1_HUMAN	ITGB1	Integrin beta-1	3	2	0.06	1.46	3.43*E* − 02
KV119_HUMAN		Ig kappa chain V-I region Wes	21	2	2.16	1.52	6.37*E* − 03
KV308_HUMAN		Ig kappa chain V-III region CLL	4	1	0.49	1.65	8.16*E* − 03
LV204_HUMAN		Ig lambda chain V-II region TRO	7	2	0.96	1.59	1.25*E* − 02
PCYOX_HUMAN	PCYOX1	Prenylcysteine oxidase 1	48	9	1.89	1.43	3.70*E* − 02
PEDF_HUMAN	SERPINF1	Pigment epithelium-derived factor	23	9	1.54	1.48	1.56*E* − 02
PON3_HUMAN	PON3	Serum paraoxonase/lactonase 3	179	12	3.89	1.51	4.11*E* − 02
Q6MZX9_HUMAN	DKFZp686M08189	Putative uncharacterized protein DKFZp686M08189	124	7	1.41	1.43	3.95*E* − 02
Q6N091_HUMAN	DKFZp686C02220	Putative uncharacterized protein DKFZp686C02220 (fragment)	71	6	1.2	1.46	2.31*E* − 02
Q6ZVX0_HUMAN		cDNA FLJ41981 fis, clone SMINT2011888, highly similar to protein Tro alpha1 H,myeloma	125	7	1.38	1.43	4.13*E* − 02
Q7Z379_HUMAN	DKFZp686K04218	Putative uncharacterized protein DKFZp686K04218 (fragment)	124	7	1.44	1.43	3.96*E* − 02
Q8TE63_HUMAN		Immunoglobulin light chain variable region (fragment)	4	2	0.97	1.45	4.51*E* − 02
Q96K68_HUMAN		cDNA FLJ14473 fis, clone MAMMA1001080, highly similar to *Homo sapiens* SNC73 protein (SNC73) mRNA	205	9	1.77	1.45	2.93*E* − 02
Q9NPP6_HUMAN		Immunoglobulin heavy chain variant (fragment)	125	7	1.75	1.43	4.92*E* − 02
Q9UL83_HUMAN		Myosin-reactive immunoglobulin light chain variable region (fragment)	14	2	2.98	1.54	5.43*E* − 03
Q9UL89_HUMAN		Myosin-reactive immunoglobulin heavy chain variable region (fragment)	18	5	8.63	1.49	2.16*E* − 02
TRPM8_HUMAN	TRPM8	Transient receptor potential cation channel subfamily M member 8	3	2	0.04	1.60	6.39*E* − 03

*Downregulated*							
A0A024QZK7_HUMAN	HK1	Hexokinase	5	1.00*E* + 00	0.03	0.82	7.31*E* − 03
A0A024R2X3_HUMAN	HYAL1	Hyaluronidase	12	5.00*E* + 00	0.55	0.73	2.82*E* − 05
A0A024R451_HUMAN	SERPINE2	Serpin peptidase inhibitor, clade E (nexin, plasminogen activator inhibitor type 1), member 2, isoform CRA_a	17	8	1.05	0.83	2.36*E* − 03
A0A024RAG6_HUMAN	C1QA	Complement component 1, q subcomponent, A chain, isoform CRA_a	12	4	1.08	0.75	4.11*E* − 04
A0A0S2Z3Y1_HUMAN	LGALS3BP	Lectin galactoside-binding soluble 3 binding protein isoform 1 (fragment)	247	14	4.17	0.71	2.49*E* − 06
A0A0S2Z4D4_HUMAN	PLP1	Proteolipid protein 1 isoform 1 (fragment)	2	1	0.11	0.40	1.65*E* − 04
ADT4_HUMAN	SLC25A31	ADP/ATP translocase 4	5	1	0.16	0.65	1.28*E* − 02
B1AHL2_HUMAN	FBLN1	Fibulin-1	74	8	0.92	0.78	4.33*E* − 04
CALU_HUMAN	CALU	Calumenin	12	7	0.86	0.76	2.45*E* − 04
CFAH_HUMAN	CFH	Complement factor H	689	31	4.02	0.83	8.87*E* − 03
LBP_HUMAN	LBP	Lipopolysaccharide-binding protein	148	11	3.18	0.79	3.74*E* − 04
LRP1_HUMAN	LRP1	Prolow-density lipoprotein receptor-related protein 1	37	20	0.16	0.83	7.44*E* − 03
LTBP1_HUMAN	LTBP1	Latent-transforming growth factor beta-binding protein 1	19	8	0.16	0.77	8.36*E* − 04
NUCB1_HUMAN	NUCB1	Nucleobindin-1	17	7	0.66	0.78	5.80*E* − 04
Q8NBH6_HUMAN		Fibulin-1	125	11	2.25	0.75	1.26*E* − 04
Q9HCC1_HUMAN		Single chain Fv (fragment)	10	2	0.96	0.79	4.29*E* − 04
THRB_HUMAN	F2	Prothrombin	1145	18	6.65	0.78	6.78*E* − 04

^a^Fold change provided by MASCOT.

^b^ *p* values calculated by edgeR to show the significance of different expression.

**Table 4:** Up- and downregulated serum proteins exclusively in DHSM-CHB.

UniProt_ID	Gene_name	Description	Number of significant matches	Number of significant sequences	emPAI	FC^a^	*p* value^b^
*Upregulated*							
A0A024R145_HUMAN	ALDOB	Fructose-bisphosphate aldolase	5	3	0.22	1.29	3.81*E* − 02
A0A024R1N1_HUMAN	MYH9	Myosin, heavy polypeptide 9, nonmuscle, isoform CRA_a	17	9	0.13	1.43	1.34*E* − 03
A0A024R5H8_HUMAN	RAB6A	RAB6A, member RAS oncogene family, isoform CRA_b	8	3	0.57	1.43	4.84*E* − 03
A0A024R694_HUMAN	ACTN1	Actinin, alpha 1, isoform CRA_a	20	10	0.42	1.33	1.97*E* − 02
A0A024R6G3_HUMAN	FBLN5	Fibulin 5, isoform CRA_b	6	3	0.27	1.33	6.31*E* − 03
A0A024R9T1_HUMAN	hCG_39634	HCG39634, isoform CRA_a	2	2	0.26	1.49	4.90*E* − 03
A0A024RDB8_HUMAN	HPSE	Heparanase, isoform CRA_a	23	6	0.55	1.36	1.03*E* − 03
A0A024RDL8_HUMAN	ASL	Argininosuccinate lyase isoform 1	10	5	0.39	1.65	2.02*E* − 07
A0A087WT59_HUMAN	TTR	Transthyretin	175	7	5.66	1.34	3.42*E* − 02
A0A087WUL0_HUMAN	TKFC	Triokinase/FMN cyclase	3	3	0.15	1.28	9.35*E* − 03
A0A0A0MSD0_HUMAN	SVEP1	Sushi, von Willebrand factor type A, EGF and pentraxin domain-containing protein 1	4	3	0.02	1.36	7.51*E* − 03
A0A0A0MT32_HUMAN	LIPA	Lysosomal acid lipase/cholesteryl ester hydrolase	2	1	0.09	1.38	4.78*E* − 03
A0A0C4DFP6_HUMAN	CRTAC1	Cartilage acidic protein 1	6	5	0.24	1.23	3.57*E* − 02
A0A0S2Z3F6_HUMAN	CETP	Cholesteryl ester transfer protein plasma isoform 1 (fragment)	95	14	3.56	1.36	1.42*E* − 03
A0A125QYY9_HUMAN		IBM-B2 heavy chain variable region (fragment)	12	2	0.83	1.42	2.25*E* − 03
A0N7I9_HUMAN	F5-20	F5-20 (fragment)	4	1	0.47	1.68	2.31*E* − 03
A2NYU9_HUMAN		Heavy chain Fab (fragment)	2	1	0.48	1.29	3.92*E* − 02
A8K486_HUMAN		Peptidyl-prolyl cis-trans isomerase	2	2	0.32	1.40	4.10*E* − 02
ADIPO_HUMAN	ADIPOQ	Adiponectin	27	5	2.13	1.32	1.12*E* − 02
APOF_HUMAN	APOF	Apolipoprotein F	7	3	0.5	1.29	1.84*E* − 02
ASSY_HUMAN	ASS1	Argininosuccinate synthase	4	3	0.18	1.49	1.09*E* − 04
ATPB_HUMAN	ATP5B	ATP synthase subunit beta, mitochondrial	11	2	0.22	1.34	3.74*E* − 02
B3KTV0_HUMAN		cDNA FLJ38781 fis, clone LIVER2000216, highly similar to HEAT SHOCK COGNATE 71 kDa PROTEIN	5	5	0.21	1.29	3.41*E* − 02
B4DQK4_HUMAN		cDNA FLJ53743, highly similar to tubulin alpha-3 chain	21	6	0.81	1.61	1.06*E* − 03
B4DVA7_HUMAN		Beta-hexosaminidase	3	2	0.15	1.26	1.84*E* − 02
B7Z478_HUMAN	PSMB2	Proteasome (prosome, macropain) subunit, beta type, 2, isoform CRA_b	2	1	0.3	1.65	1.96*E* − 03
BPIB1_HUMAN	BPIFB1	BPI fold-containing family B member 1	11	6	0.51	1.28	1.34*E* − 02
BTD_HUMAN	BTD	Biotinidase	47	6	1.12	1.54	1.69*E* − 06
CAMP_HUMAN	CAMP	Cathelicidin antimicrobial peptide	2	1	0.29	1.43	2.64*E* − 03
CAND1_HUMAN	CAND1	Cullin-associated NEDD8-dissociated protein 1	2	1	0.02	1.36	2.60*E* − 03
CATD_HUMAN	CTSD	Cathepsin D	2	1	0.13	1.28	3.06*E* − 02
DHSO_HUMAN	SORD	Sorbitol dehydrogenase	4	3	0.32	1.67	2.46*E* − 07
FIBA_HUMAN	FGA	Fibrinogen alpha chain	102	22	2.36	1.36	3.18*E* − 03
G3P_HUMAN	GAPDH	Glyceraldehyde-3-phosphate dehydrogenase	19	7	1.37	1.37	5.07*E* − 03
G3V5Z7_HUMAN	PSMA6	Proteasome subunit alpha type	7	4	0.58	1.44	9.01*E* − 04
HV310_HUMAN		Ig heavy chain V-III region HIL	3	1	0.5	1.48	1.12*E* − 03
HV320_HUMAN		Ig heavy chain V-III region GAL	36	3	1.91	1.32	2.19*E* − 02
KV404_HUMAN		Ig kappa chain V-IV region B17	34	2	1.45	1.45	4.98*E* − 04
LDHB_HUMAN	LDHB	L-lactate dehydrogenase B chain	4	2	0.33	1.65	2.13*E* − 06
M0QZB5_HUMAN	PPFIA4	Liprin-alpha-4 (fragment)	2	1	0.03	1.33	2.70*E* − 02
MARCO_HUMAN	MARCO	Macrophage receptor MARCO	2	1	0.11	1.31	2.89*E* − 02
MDR3_HUMAN	ABCB4	Phosphatidylcholine translocator ABCB4	2	1	0.04	1.32	1.23*E* − 02
OTUB1_HUMAN	OTUB1	Ubiquitin thioesterase OTUB1	2	1	0.09	1.27	2.92*E* − 02
PIGR_HUMAN	PIGR	Polymeric immunoglobulin receptor	7	4	0.18	1.34	4.27*E* − 03
PSA3_HUMAN	PSMA3	Proteasome subunit alpha type-3	8	4	0.58	1.31	4.84*E* − 02
PSB1_HUMAN	PSMB1	Proteasome subunit beta type-1	4	3	0.5	1.47	1.25*E* − 03
Q6GMX4_HUMAN	IGL@	IGL@ protein	442	6	7.15	1.27	3.98*E* − 02
Q6PIQ7_HUMAN	IGL@	IGL@ protein	441	6	6.39	1.27	4.86*E* − 02
Q6ZNX5_HUMAN		CDNA FLJ26936 fis, clone RCT06808	5	1	0.39	1.78	2.10*E* − 08
Q9UL82_HUMAN		Myosin-reactive immunoglobulin light chain variable region (fragment)	46	3	2.11	1.41	1.22*E* − 03
QSOX1_HUMAN	QSOX1	Sulfhydryl oxidase 1	97	20	2.33	1.24	4.15*E* − 02
TBA4A_HUMAN	TUBA4A	Tubulin alpha-4A chain	24	8	0.86	1.55	1.38*E* − 03
TBB1_HUMAN	TUBB1	Tubulin beta-1 chain	6	4	0.33	1.30	2.87*E* − 02
TBB8_HUMAN	TUBB8	Tubulin beta-8 chain	7	5	0.42	1.32	3.10*E* − 02
TRML1_HUMAN	TREML1	Trem-like transcript 1 protein	3	1	0.09	1.42	1.05*E* − 03
TSP1_HUMAN	THBS1	Thrombospondin-1	365	34	4.23	1.37	1.66*E* − 03
TYPH_HUMAN	TYMP	Thymidine phosphorylase	3	3	0.19	1.23	4.67*E* − 02

*Downregulated*							
A0A024CIM4_HUMAN		Carboxylic ester hydrolase	7	3	0.17	0.81	1.43*E* − 02
A0A024R6I6_HUMAN	SERPINA10	Serpin peptidase inhibitor, clade A (alpha-1 antiproteinase, antitrypsin), member 10, isoform CRA_b	65	12	2.73	0.75	1.46*E* − 04
A0A024R6K8_HUMAN	WARS	Tryptophanyl-tRNA synthetase, isoform CRA_a	5	2	0.1	0.82	4.87*E* − 02
A0A024R853_HUMAN	IQCE	IQ motif containing E, isoform CRA_b	2	1	0.04	0.74	1.67*E* − 03
A0A024R9J3_HUMAN	COLEC10	Collectin subfamily member 10 (C-type lectin), isoform CRA_a	4	2	0.28	0.83	2.40*E* − 02
A0A075B6S2_HUMAN	IGKV2D-29	Protein IGKV2D-29 (fragment)	26	3	2.47	0.77	2.05*E* − 03
A0A087X054_HUMAN	HYOU1	Hypoxia upregulated protein 1	4	2	0.08	0.58	2.93*E* − 08
A0A0A7C3P2_HUMAN	HLA-A	MHC class I antigen (fragment)	8	3	0.72	0.77	1.24*E* − 03
A0A0J9YX35_HUMAN		Uncharacterized protein (fragment)	2	1	0.51	0.74	3.43*E* − 03
A0A0X9TD47_HUMAN		MS-D1 light chain variable region (fragment)	30	3	5.49	0.83	1.31*E* − 02
A2AP_HUMAN	SERPINF2	Alpha-2-antiplasmin	67	10	1.86	0.74	2.85*E* − 04
A2J1N4_HUMAN		Rheumatoid factor RF-IP24 (fragment)	10	2	1.08	0.80	4.59*E* − 03
A2J1N5_HUMAN		Rheumatoid factor RF-ET6 (fragment)	15	1	1.65	0.79	6.40*E* − 04
A2MYD0_HUMAN	V1-17	V1-17 protein (fragment)	8	3	1.87	0.83	1.21*E* − 02
A2NB45_HUMAN		Cold agglutinin FS-1 L-chain (fragment)	23	2	1.39	0.69	3.83*E* − 04
A3RKG7_HUMAN		Coagulation factor VII (fragment)	17	6	2.02	0.77	7.55*E* − 04
ALBU_HUMAN	ALB	Serum albumin	1841	31	17.82	0.67	1.64*E* − 06
AMBP_HUMAN	AMBP	Protein AMBP	34	3	0.75	0.76	4.96*E* − 04
ATS13_HUMAN	ADAMTS13	A disintegrin and metalloproteinase with thrombospondin motifs 13	22	8	0.26	0.81	6.52*E* − 03
B2RBZ5_HUMAN		cDNA, FLJ95778, highly similar to *Homo sapiens* serpin peptidase inhibitor, clade A (alpha-1 antiproteinase, antitrypsin), member 10 (SERPINA10), mRNA	58	11	2.2	0.76	1.77*E* − 04
B4DL32_HUMAN		cDNA FLJ59922, highly similar to keratin, type II cytoskeletal 5	7	2	0.33	0.83	2.87*E* − 02
B4DN21_HUMAN		cDNA FLJ53365, highly similar to *Homo sapiens* fibronectin 1 (FN1), transcript variant 4, mRNA	384	8	6.85	0.77	1.76*E* − 03
B4E1B2_HUMAN		cDNA FLJ53691, highly similar to serotransferrin	142	16	2.78	0.59	6.58*E* − 09
B7Z8B6_HUMAN		cDNA FLJ54395, highly similar to inter-alpha-trypsin inhibitor heavy chain H1	68	8	1.2	0.71	6.32*E* − 06
BIG3_HUMAN	ARFGEF3	Brefeldin A-inhibited guanine nucleotide-exchange protein 3	3	1	0.01	0.71	7.55*E* − 03
BLVRB_HUMAN	BLVRB	Flavin reductase (NADPH)	6	2	0.28	0.81	1.46*E* − 02
CATA_HUMAN	CAT	Catalase	3	3	0.15	0.80	4.91*E* − 03
CATF_HUMAN	CTSF	Cathepsin F	6	4	0.23	0.81	2.58*E* − 03
CO2_HUMAN	C2	Complement C2	20	9	0.5	0.78	1.25*E* − 03
CO5_HUMAN	C5	Complement C5	416	52	4.64	0.74	4.50*E* − 04
F13B_HUMAN	F13B	Coagulation factor XIII B chain	4	3	0.15	0.76	1.83*E* − 03
GLUC_HUMAN	GCG	Glucagon	2	1	0.3	0.81	8.52*E* − 03
H0YLF3_HUMAN	B2M	Beta-2-microglobulin (fragment)	4	1	0.75	0.77	5.98*E* − 03
HEMO_HUMAN	HPX	Hemopexin	63	9	1.51	0.65	4.59*E* − 08
HGFA_HUMAN	HGFAC	Hepatocyte growth factor activator	15	4	0.39	0.82	4.99*E* − 03
HV306_HUMAN		Ig heavy chain V-III region BUT	36	5	6.45	0.76	2.60*E* − 04
HV314_HUMAN		Ig heavy chain V-III region LAY	3	2	0.88	0.81	8.62*E* − 03
HV317_HUMAN		Ig heavy chain V-III region ZAP	3	1	0.56	0.78	1.26*E* − 03
IC1_HUMAN	SERPING1	Plasma protease C1 inhibitor	34	7	0.82	0.62	1.32*E* − 09
INHBC_HUMAN	INHBC	Inhibin beta C chain	16	3	0.36	0.82	1.47*E* − 02
ITIH1_HUMAN	ITIH1	Inter-alpha-trypsin inhibitor heavy chain H1	252	22	3	0.81	1.20*E* − 02
K1C10_HUMAN	KRT10	Keratin, type I cytoskeletal 10	32	8	0.87	0.70	1.75*E* − 06
K1C14_HUMAN	KRT14	Keratin, type I cytoskeletal 14	3	2	0.11	0.68	1.74*E* − 06
K1C9_HUMAN	KRT9	Keratin, type I cytoskeletal 9	24	8	0.65	0.81	5.29*E* − 03
K22E_HUMAN	KRT2	Keratin, type II cytoskeletal 2 epidermal	38	14	1.31	0.77	4.04*E* − 04
K2C1_HUMAN	KRT1	Keratin, type II cytoskeletal 1	104	19	4.31	0.66	1.81*E* − 08
K2C6B_HUMAN	KRT6B	Keratin, type II cytoskeletal 6B	18	4	0.51	0.63	3.76*E* − 09
K7ER74_HUMAN	APOC4-APOC2	Protein APOC4-APOC2	111	5	7.56	0.68	7.94*E* − 05
KV201_HUMAN		Ig kappa chain V-II region Cum	23	2	1.98	0.54	2.24*E* − 09
L0R8K6_HUMAN	TRIM25	Alternative protein TRIM25	13	1	0.9	0.77	8.84*E* − 04
MASP1_HUMAN	MASP1	Mannan-binding lectin serine protease 1	36	7	0.63	0.83	1.78*E* − 02
PCDGG_HUMAN	PCDHGB4	Protocadherin gamma-B4	3	1	0.03	0.80	4.10*E* − 02
PLA1A_HUMAN	PLA1A	Phospholipase A1 member A	2	1	0.07	0.83	5.39*E* − 03
PROC_HUMAN	PROC	Vitamin K-dependent protein C	84	9	1.71	0.78	1.40*E* − 03
Q06AH7_HUMAN	TF	Transferrin	141	16	2.64	0.59	6.40*E* − 09
Q1T720_HUMAN	HLA-B	MHC class I antigen (fragment)	9	6	0.9	0.77	1.30*E* − 03
Q6U2L6_HUMAN	C4B	C4B (fragment)	79	6	27.02	0.69	2.60*E* − 05
Q9P1C5_HUMAN		PRO2769	43	7	0.97	0.70	1.53*E* − 06
Q9UMV1_HUMAN	C4B	Complement C4B1a (fragment)	8	1	1.56	0.77	3.10*E* − 04
Q9UNU2_HUMAN	C4B	Complement protein C4B frameshift mutant (fragment)	524	14	26.83	0.64	8.08*E* − 07
QPCT_HUMAN	QPCT	Glutaminyl-peptide cyclotransferase	7	4	0.43	0.83	1.22*E* − 02
TIMP3_HUMAN	TIMP3	Metalloproteinase inhibitor 3	2	1	0.11	0.75	1.01*E* − 03

^a^Fold change provided by MASCOT.

^b^ *p* values calculated by edgeR to show the significance of different expression.
